# Using mobility status as a frailty indicator to improve the accuracy of a computerised five-level triage system among older patients in the emergency department

**DOI:** 10.1186/s12873-022-00646-0

**Published:** 2022-05-19

**Authors:** Cheng-Yu Chien, Chung-Hsien Chaou, Chung-Cheng Yeh, Kuang-Hung Hsu, Shi-Ying Gao, Chip-Jin Ng

**Affiliations:** 1 Department of Emergency Medicine, Chang Gung Memorial Hospital, Linkou and College of Medicine, Chang Gung University, No. 5 Fushing St., Gueishan Dist, Taoyuan City, 333 Taiwan; 2 Department of Emergency Medicine, Ton-Yen General Hospital, Zhubei, 302 Taiwan; 3 Graduate Institute of Management, Chang Gung University, Taoyuan, 333 Taiwan; 4 Institute of Epidemiology and Preventive Medicine, College of Public Health, National Taiwan University, Taipei, 100 Taiwan; 5 Chang Gung Medical Education Research Center, Chang Gung Memorial Hospital, Linkou, Taoyuan, 333 Taiwan; 6 Department of Emergency Medicine, Chang Gung Memorial Hospital, Keelung Branch, Keelung, 204 Taiwan; 7 Laboratory for Epidemiology, Department of Health Care Management, Healthy Aging Research Center, Chang Gung University, Taoyuan, 333 Taiwan; 8 Research Center for Food and Cosmetic Safety, College of Human Ecology, Chang Gung University of Science and Technology, Taoyuan, 333 Taiwan; 9 Department of Safety, Health and Environmental Engineering, Ming Chi University of Technology, New Taipei City, 243 Taiwan; 10 Department of Emergency Medicine, Chang Gung Memorial Hospital, Linkou, Taoyuan, 333 Taiwan

**Keywords:** Frailty, Older patients, Triage acuity scale, Emergency department

## Abstract

**Background:**

Owing to societal ageing, the number of older individuals visiting emergency departments (EDs) has increased in recent years. For this patient population, accurate triage systems are required. This retrospective cohort study assessed the accuracy of a computerised five-level triage system, the Taiwan Triage and Acuity System (TTAS), by determining its ability to predict in-hospital mortality in older adult patients and compare it with the corresponding rate in younger adult patients presenting to EDs. The association between frailty, which the current triage system does not consider, was also investigated.

**Methods:**

The medical records of adult patients admitted to a single ED between 2016 and 2017 were reviewed. Data collected included information on demographics, triage level, frailty status, in-hospital mortality, and medical resource utilisation. The patients were divided into four age groups: two older adult groups (older: 65–84 years and very old: ≥85 years) and two younger adult groups (young: 18–39 and middle-aged: 40–64 years).

**Results:**

Our study included 265,219 ED adult patients, of whom 64,104 and 16,009 were in the older and very old groups, respectively. The in-hospital mortality rate at each triage level increased with age. The ability of the TTAS to predict in-hospital mortality decreased with age (area under the receiver operating characteristic curve [AUROC]: young: 0.86; middle-aged, 0.84; and older and very old: 0.79). Frailty was associated with in-hospital mortality (odds ratio, 2.20; 95% confidence interval, 2.03–2.38). Adding mobility status as a frailty indicator to TTAS only slightly improved its ability to predict in-hospital mortality (AUROC: 0.74–0.77) in patients ≥65 years of age.

**Conclusions:**

The ability of the current triage system to predict in-hospital mortality decreases with age. Although frailty as mobility was associated with in-hospital mortality, its addition to the TTAS only slightly improved the accuracy with which in-hospital mortality in older patients presenting to EDs was predicted.

**Supplementary Information:**

The online version contains supplementary material available at 10.1186/s12873-022-00646-0.

## Introduction

Population aging imposes burdens on society and healthcare systems. Emergency departments (EDs) are among the affected entities; the number of ED visits by older individuals (those aged ≥65 years [1]) increased by 40% between 2002 and 2012 [2]. Such visits accounted for 17 and 21% of all ED visits in Korea [3] and Taiwan [4], respectively, in recent years.

The first vital step towards improving overall ED care for older patients is optimising triage systems. However, atypical presentations of common diseases (e.g., acute coronary syndrome and acute stroke) and cognitive issues make it challenging to accurately triage and prioritise the older patients [5]. Although factors such as comorbidities, polypharmacy, and palliative care are clearly related to in-hospital mortality [6, 7] in older patients, they are challenging to assess during triage assignment in the frequently busy and overcrowded ED.

To identify and uptriage the older ED patients who are most likely to deteriorate while waiting to be seen by a physician, a frailty modifier was added to the Canadian Triage and Acuity Scale (CTAS) [8]. Frailty is a multifactorial syndrome characterised by a heightened vulnerability to adverse health events and a diminished physiologic reserve, inhibiting homeostatic recovery from stressors [9]. Functional decline and disability often manifest in falls and difficulties with mobility [10].

The Taiwan Triage and Acuity Scale (TTAS), a validated computerised five-level triage system adapted from the CTAS, serves as the standard emergency triage system in Taiwan [11]. Triage severity is determined based on chief complaints, vital signs, and pain scores [12], but unlike the revised CTAS, does not consider frailty. In view of societal ageing [13], assessing the accuracy of the TTAS in application to older patients is crucial. Accordingly, we examined the ability of the TTAS to predict in-hospital mortality in older adult patients and compared it with the corresponding rate in younger adult patients. In view of the evidence that mobility status is one of the phenotypes of frailty in older adults [14, 15], we also examined the association between frailty as measured by mobility status and in-hospital mortality.

## Methods

### Study setting and population

This retrospective cohort study used deidentified data retrieved from the medical records of the ED of Linkou Chang Gung Memorial Hospital (Taoyuan City, Taiwan), which are stored in the Chang Gung Research Database. Approximately 180,000 ED visits are made annually to the ED of Linkou Chang Gung Memorial Hospital, a Joint Commission International-accredited, multispecialty medical institution with 3600 beds and 29 specialty centres. The study included patients aged ≥18 years who presented to the ED between January 2016 and December 2017. The study protocol was approved by the Institutional Review Board of our hospital (approval number: 202000012B0).

### Data collection

Patient demographic data (e.g., sex and age), clinical characteristics (e.g., vital signs, triage level, pain score), chief complaints, and ED disposition were extracted from the database.

In the TTAS, triage levels are categorised as follows: level 1, resuscitation; level 2, emergency; level 3, urgent; level 4, less urgent; and level 5, nonurgent. Patients are evaluated and then assigned a triage level by a triage nurse [12].

Considering the time constraints in triage, mobility was employed as a frailty indicator in this study because it can be easily and rapidly assessed. Patients were categorised as nonfrail or frail using the mobility category of the ‘Risk of Adult Falling Form’ (see supplemental file). The fall risk assessment form, modified from the Morse Fall Scale [15] by local experts, has been used as a routine nursing assessment for more than 15 years at our institution. Subjects with a total score of 5 or more were indicated as “Frailty”. Generally, patients were considered nonfrail when they appeared normal, fit, or well-managed and were considered frail when they had difficulty balancing, had an unsteady gait, used a mobility aid, or had poor lower limb muscle strength.

Conventionally, older people refers to individuals aged 65 years and older. In this study, older adults were divided into two groups (older [65–84 years] and very old [≥85 years] groups) [3] and compared with younger adults (young [18–39 years and middle-aged [40–64 years] groups).

### Outcomes

The primary outcome was in-hospital mortality. The secondary outcome was medical resource utilisation, as determined by the number of Taiwan National Health Insurance (NHI) claim points. Since the implementation of NHI in 1995, the payment methods are principally fee-for-services based for both outpatient and inpatient service. Furthermore, NHI also keeps a complete database which is open to the public upon application [16]. As a result, medical resource utilisation can be indicated by the sum of these NHI clam points. Medical resource utilisation was defined as the medical management costs incurred in the first 6 hours after ED arrival to avoid overestimation due to prolonged ED stay [11].

### Statistical analysis

Categorical variables are presented as numbers and percentages and were compared using the chi-squared test or Fisher’s exact test, as appropriate. Continuous variables are presented as means and standard deviations or as medians and ranges. The student’s *t*-test and the Mann–Whitney *U* test were conducted on normally and nonnormally distributed continuous variables, respectively. Univariable and multivariable logistic regression models were used to identify the variables associated with in-hospital mortality. The multivariable logistic regression model was assessed by the likelihood ratio test (Cox Snell R^2^ = 0.3030, *p* < 0.001). The odds ratio (OR) is reported with a 95% confidence interval (CI). The area under the receiver operating characteristic curve (AUROC) was determined to evaluate the ability of the TTAS to predict in-hospital mortality and medical resource utilisation among younger and older patients. The available case analysis method was used to handle data not missing at random. The total number of observation (n) of each variable was presented. A *p-*value < 0.05 was considered statistically significant. Analyses were conducted using SPSS Statistics for Windows, version 13.0 (SPSS Inc., Chicago, IL, USA).

## Results

**Fig. 1:**
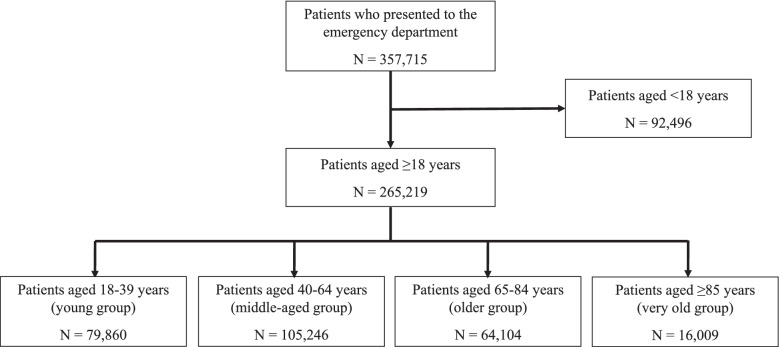
Flow diagram of patient enrollment

A total of 357,715 patients presented to the ED from January 2016 to December 2017. Of these patients, 265,219 individuals were aged ≥18 years and were included in the present study. They were divided into four age groups: young (*n* = 79,860), middle-aged (*n* = 105,246), older (*n* = 64,104), and very old (*n* = 16,009; Fig. 1).

Overall, 51.6% of the ED patients were men, and more than half (67.4%) were triaged as level 3 (Table 1). Significant differences were observed in all baseline characteristics across the age groups. The proportion of ED patients triaged as level 1 or 2 increased significantly with age, whereas the proportion of ED patients triaged as levels 3–5 decreased significantly with age. The percentage of ED patients who were identified as frail, experienced in-hospital mortality, and used medical resources also increased significantly with age. Shortness of breath was more common in the older (8.4%) and very old (13.4%) patients than in the younger patients (3.8%). The chief complaints of the older and very old adults, but not of the younger adults, included generalised weakness, device issues, and bloody stool (see supplementary Table 1).

**Table 1:** Baseline characteristics of the patients

	Total (*N* = 265,219)	Age group				*p*-value
		18–39 (*N* = 79,860)	40–64 (*N* = 105,246)	65–84 (*N* = 64,104)	≥85 (*N* = 16,009)	
Male (*n* = 265,218)	136,776 (51.6)	37,984 (47.6)	57,977 (55.1)	32,687 (50.1)	8128 (50.8)	< 0.001
Age, years*	52.95 (19.7)	29.57 (6.10)	52.61 (7.22)	73.66 (5.84)	88.91 (3.45)	< 0.001
Triage level						< 0.001
1	12,518 (4.72)	2722 (3.41)	3487 (3.31)	4474 (6.98)	1835 (11.5)	
2	46,877 (17.7)	7847 (9.83)	17,575 (16.7)	16,009 (25.0)	5446 (34.0)	
3	178,857 (67.4)	57,515 (72.0)	73,991 (70.3)	39,346 (61.4)	8005 (50.0)	
4	25,276 (9.53)	11,084 (13.9)	9494 (9.02)	4009 (6.25)	689 (4.30)	
5	1691 (0.64)	692 (0.87)	699 (0.66)	266 (0.41)	34 (0.21)	
Frailty (*n* = 121,084)						< 0.001
No	107,917 (89.1)	33,621 (96.2)	44,955 (92.3)	24,444 (81.5)	4897 (66.1)	
Yes	13,167 (10.9)	1337 (3.82)	3779 (7.75)	5539 (18.5)	2512 (33.9)	
Vital signs						
Pulse rate, beats/min* (*n* = 265,087)	90.36 (20.1)	92.11 (19.3)	90.77 (19.8)	88.19 (20.8)	87.64 (21.5)	< 0.001
SBP, mmHg* (*n* = 264,235)	140.37 (29.4)	131.90 (21.5)	142.92 (29.9)	146.11 (33.3)	142.84 (34.1)	< 0.001
DBP, mmHg* (*n* = 264,188)	84.34 (37.4)	86.68 (38.6)	87.57 (36.9)	81.57 (36.1)	77.55 (37.5)	< 0.001
Respiratory rate, times/min* (*n* = 265,068)	18.91 (2.77)	18.43 (1.92)	18.82 (2.65)	19.38 (3.32)	20.08 (3.92)	< 0.001
Body temperature, °C* (*n* = 265,115)	36.60 (1.06)	36.66 (0.91)	36.56 (1.02)	36.59 (1.22)	36.60 (1.31)	< 0.001
Blood oxygen saturation, %* (*n* = 260,369)	95.85 (5.51)	96.80 (3.19)	96.08 (5.01)	94.90 (6.99)	93.67 (8.61)	< 0.001
Pain score* (*n* = 237,824)	1.34 (1.89)	1.58 (1.93)	1.45 (1.98)	1.01 (1.71)	0.82 (1.53)	< 0.001
GCS (*n* = 265,218)						< 0.001
14–15	249,674 (94.1)	78,595 (98.4)	101,065 (96.0)	57,591 (89.8)	12,423 (77.6)	
9–13	9559 (3.60)	706 (0.88)	2548 (2.42)	4063 (6.34)	2242 (14.0)	
≤ 8	5985 (2.26)	558 (0.70)	1633 (1.55)	2450 (3.82)	1344 (8.40)	
Time of ED visit						< 0.001
08:00–16:00	116,412 (43.9)	28,713 (36.0)	4644 (44.2)	32,774 (51.1)	8441 (52.7)	
16:00–24:00	103,049 (38.9)	33,776 (42.3)	40,590 (38.6)	22,977 (35.8)	5706 (35.6)	
00:00–08:00	45,758 (17.3)	17,371 (21.8)	18,172 (17.3)	8353 (13.0)	1862 (11.6)	
Outcomes						
In-hospital mortality (*n* = 260,741)	6819 (2.62)	343 (0.44)	2426 (2.35)	2830 (4.48)	1220 (7.69)	< 0.0001
Medical resource utilisation* (*n* = 132,558)	6.62 (15.4)	4.18 (19.6)	6.77 (12.5)	8.81 (14.00)	9.48 (12.7)	< 0.001

Data are presented as n (%) or mean (SD). Data presented as mean (SD) are marked with asterisk*

*Medical resource utilisation as Taiwan National Health Insurance claim points

*Abbreviations*: *N* number of subjects, *SD* Standard deviation, *SBP* Systolic blood pressure, *DBP* Diastolic blood pressure, *min* Minute, *GSC* Glasgow Coma Scale, *ED* Emergency department

As presented in Table 2, the young adults had the lowest in-hospital mortality rate and very old adults had the highest in-hospital mortality rate across TTAS levels. The AUROC for in-hospital mortality was 0.86 (95% CI, 0.84–0.88) in the young adults and decreased to 0.79 (95% CI, 0.78–0.80) in the very old adults. For medical resource utilisation, the middle-aged group had the highest utilisation compared with the other age groups in level 1 or 2, followed by the older and very old groups. The AUROC for medical resource utilisation was 0.66 (95% CI, 0.66–0.67) in the young adults and increased to 0.74 (95% CI, 0.73–0.75) in the very old adults.

**Table 2:** Summary of in-hospital mortality and medical resource utilisation by triage level and their predictability (AUROC) by current triage system across age groups. The TTAS was the triage system current in used which frailty assessment was not incorporated

	Total	Age group			
		18–39	40–64	65–84	≥85
In-hospital mortality rate by TTAS level, n/N1 (%)					
1	2769/12,445 (22.3)	169/2670 (6.33)	942/3477 (27.1)	1106/4465 (24.8)	552/1833 (30.1)
2	2803/46,751 (6)	103/7803 (1.32)	997/17,526 (5.69)	1164/15,982 (7.28)	539/5440 (9.91)
3	1222/175,678 (0.7)	68/56,545 (0.12)	479/72,567 (0.66)	549/38,672 (1.42)	126/7894 (1.60)
4	24/24,315 (0.1)	3/10,712 (0.03)	7/9085 (0.08)	11/3848 (0.29)	3/670 (0.45)
5	1/1552 (0.06)	0/636 (0)	1/637 (0.16)	0/247 (0)	0/32 (0)
AUROC (95% CI)	0.838 (0.833–0.843)	0.862 (0.841–0.883)	0.840 (0.832–0.848)	0.792 (0.784–0.801)	0.794 (0.783–0.806)
Medical resource utilisation^a^ by TTAS level, mean (SD)					
1	15.1 (19.36)	8.34 (17.39)	18.1 (23.13)	16.74 (18.56)	14.41 (11.86)
2	12.69 (29.91)	9.25 (59.05)	13.68 (20.43)	13.26 (18.41)	12.78 (16.49)
3	4.94 (7.26)	3.81 (5.4)	5.04 (7.64)	6.19 (8.47)	6.45 (7.91)
4	3.52 (9.88)	2.00 (2.73)	3.67 (10.19)	6.99 (17.25)	5.16 (12.96)
5	2.29 (13.74)	1.85 (2.71)	1.89 (4.66)	4.57 (34.04)	2.28 (3.57)
AUROC^b^ (95% CI)	0.715 (0.712–0.718)	0.661 (0.656–0.667)	0.706 (0.702–0.710)	0.714 (0.709–0.719)	0.738 (0.728–0.748)

Refer to Table 1 for the number of subjects with in-hospital mortality and medical resource utilisation within each age group

^a^Medical resource utilisation as Taiwan National Health Insurance claim points

^b^For AUROC analysis, medical resource utilisation was dichotomised to binary outcome using the mean medical resource utilisation (6.62, refer Table 1) in the total population as cutoff

*Abbreviations*: *n* Number of subjects with in-hospital mortality at a specified TTAS level, *N1* Number of subjects at a specified TTAS level, *AUROC* Area under the receiver operating characteristic curve, *CI* Confidence interval, *SD* Standard deviation

In the multivariable logistic regression analysis (Table 3), sex, age, TTAS level, frailty status, and time of ED visit were significantly associated with in-hospital mortality. The in-hospital mortality rate among the very old adults (OR, 5.3; 95% CI, 4.4–6.4) was significantly higher than that among the young adults. ED patients categorised as level 1 or 2 (OR, 8.6; 95% CI, 7.9–9.4) or identified as frail (OR, 2.2; 95% CI, 2.0–2.4) were significantly more likely to experience in-hospital mortality than those with other triage levels or those identified as nonfrail, respectively.

**Table 3:** Univariable and multivariable regression models on in-hospital mortality

Variables	Univariable		Multivariable	
	Odds ratio (95% CI)	*p*-value	Odds ratio (95% CI)	*p*-value
Male sex	1.55 (1.47–1.62)	< 0.001	1.32 (1.22–1.42)	< 0.001
Age group				
18–39	Reference		Reference	
40–64	5.47 (4.88–6.13)	0.019	3.33 (2.83–3.93)	< 0.001
65–84	10.66 (9.53–11.93)	< 0.001	4.27 (3.62–5.03)	< 0.001
≥ 85	18.94 (16.78–21.38)	< 0.001	5.32 (4.44–6.36)	< 0.001
TTAS				
1–2	14.83 (13.93–15.79)	< 0.001	8.62 (7.87–9.44)	< 0.001
3	Reference		Reference	
4–5	0.14 (0.09–0.21)	< 0.001	0.21 (0.13–0.33)	< 0.001
Frailty				
Yes	5.74 (5.34–6.16)	< 0.001	2.20 (2.03–2.38)	< 0.001
No	Reference		Reference	
Time of ED visit				
08:00–16:00	1.39 (1.29–1.49)	< 0.001	1.15 (1.04–1.28)	< 0.001
16:00–24:00	1.11 (1.03–1.19)	0.026	0.96 (0.85–1.07)	0.004
00:00–08:00	Reference		Reference	

*Abbreviation*: *CI* Confidence interval, *TTAS* Taiwan Triage and Acuity Scale, *ED* Emergency department

**Fig. 2:**
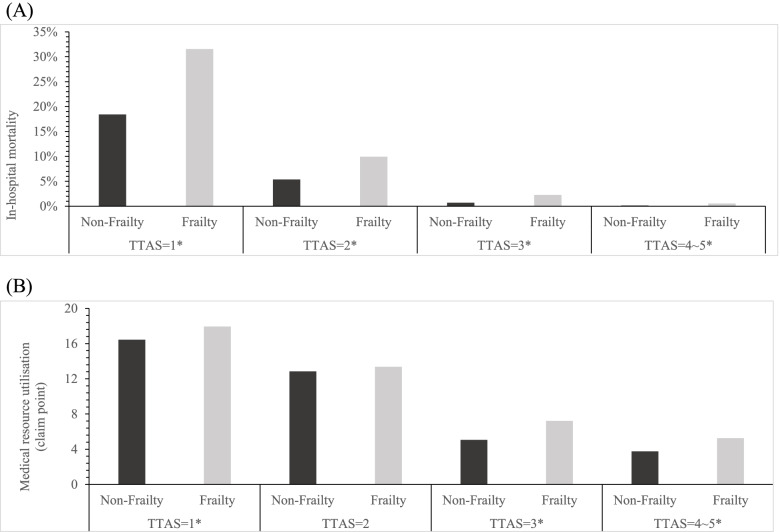
The **A** in-hospital mortality rate and **B** medical resource utilization for patients stratified by frailty status and TTAS level. * indicated *p*-value < 0.001

**Fig. 3:**
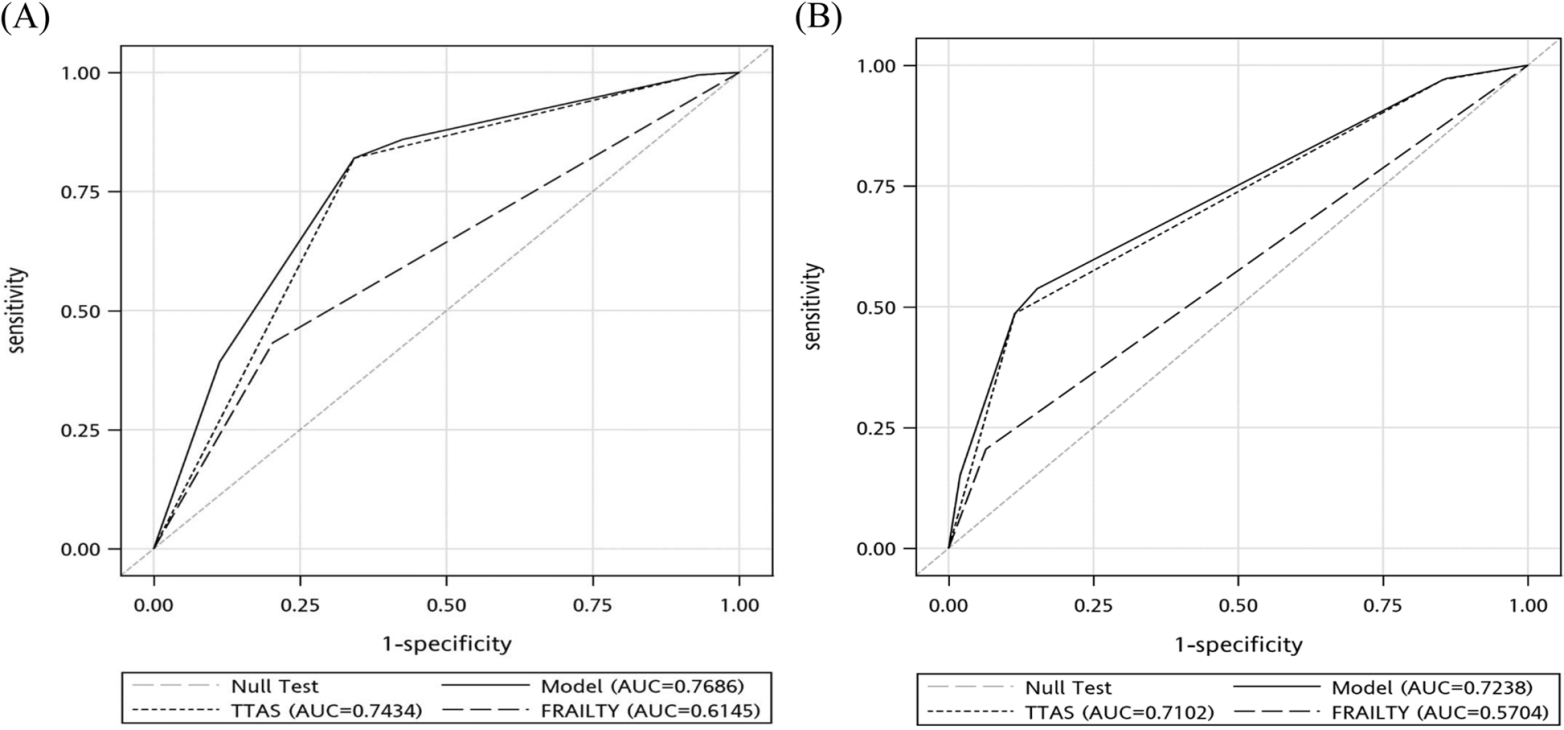
Receiver operating characteristics curves of **A** in-hospital mortality or **B** medical resource utilisation and frailty (dashed line), current TTAS (dotted line), and model (TTAS with frailty; solid line) in older patients ≥65 years

ED patients identified as frail had a significantly higher in-hospital mortality rate than those identified as nonfrail across TTAS levels (Fig. 2A). They also used significantly more medical resources than their counterparts across TTAS levels, except those at level 2 (Fig. 2B). However, adding mobility a frailty indicator to TTAS did not lead to a considerable improvement in the ability to predict in-hospital mortality (AUROC: 0.74–0.77) and medical resource utilisation (AUROC: 0.71–0.72) in patients aged ≥65 years (Fig. 3A and B).

## Discussion

Our study used a validated electronic health record system with a large sample size to determine whether the TTAS accurately triages the growing number of older patients presenting to EDs or whether a new or adjusted system is required. The predictions of the TTAS regarding in-hospital mortality were less accurate for older adults than for younger adults. Moreover, frailty was independently and positively associated with in-hospital mortality across TTAS levels. However, adding mobility status as a frailty indicator to the triage system did not lead to a considerable improvement in the ability of the TTAS to predict in-hospital mortality in older adults.

Triage systems are typically designed to screen all patients evenly; in other words, younger and older adults undergo the same screening process. This is potentially problematic because age is a potential modifier in triage; it is strongly associated with greater mortality risk in triage patients [17] and independently predicts hospital admission in patients aged > 65 years who are triaged as level 5 [18]. Herein, the in-hospital mortality rate increased significantly with age. We also observed that the young adults had the lowest in-hospital mortality rate of 6.3%, which was almost one-fourth of the average in-hospital mortality rate of patients triaged as level 1 under the TTAS. This may be due to the faster recovery rates of this age group following immediate management.

Frailty greatly differs between older and younger patients and is a problematic manifestation of population ageing. It is a consequence of the age-related deterioration of multiple physiological systems, which renders patients vulnerable to sudden changes in health status triggered by relatively minor stressors [19]. Blomaard et al. reported that the 30-day mortality rate in older high-risk individuals was three times higher than in low-risk individuals, suggesting that a modifier for the screening of older adults would improve the accuracy with which this population is triaged [20]. By contrast, Mowbray et al. noted that triage acuity and frailty were independent but complementary measures with distinct clinical outcomes in patients aged ≥75 years [9]. In the present study, mobility status as a frailty indicator was independently associated with in-hospital mortality but did not enhance the ability of the TTAS to predict in-hospital mortality in older patients. The present study used mobility as an indicator for the rapid determination of frailty status. Future studies may use more comprehensive frailty assessments such as the clinical frailty scale [21, 22] or geriatric screening tools such as the Acutely Presenting Older Patient screener [20], and to confirm their usefulness as triage tools in the ED.

As expected in our study, the older the patients, the higher the medical resource utilisation. However, Unlike the younger population, the elders often have more comorbidities and are usually weaker. Older patients and or their caregivers tend to have DNR or choose not to undergo unwanted or life-extending treatments, and this is a possible reason for the observation. In addition, the older adults cannot explain or express themselves clearly than young age group when coming into a critical illness, leading to the problem of under-diagnose and the under-estimate. Thus, current five-level triage system may have the problem of under-triage in the older populatuon. Therefore, we propose using mobility status as frailty indicator to improve the five-level triage system to better evaluate the older adults in EDs.

The capacity of triage systems to predict in-hospital mortality appears to decline with patient age. Such systems include the Japan Acuity and Triage Scale (AUROC: 0.74, 0.69, and 0.66 in older adults aged 65–74, 75–84, and ≥ 85 years, respectively) [23] and the Manchester triage system (AUROC: 0.79 and 0.71 in younger adults and older adults, respectively) [24]. In our study, the AUROC corresponding to the TTAS’s prediction of in-hospital mortality was 0.84 in all ED adult patients, 0.86 in the young adults, and 0.79 in the older and very old adults. Although the TTAS presented an acceptable ability to predict this outcome (AUROC > 0.70), the AUROC decreased with patient age, suggesting that the room for improvement remains with regard to the prediction accuracy of the TTAS for older patients. We therefore recommend a revision to the current TTAS to triage older patients more accurately. However, whether the addition of a frailty modifier (as in the case of the CTAS) is advisable remains to be determined, as does which frailty assessment should be incorporated.

### Limitations

Our study has some limitations. First, the data were extracted from a single medical centre. Although our centre is one of the largest in Taiwan and has a high ED volume, our findings may not be generalisable to other settings, regions, or countries. Second, we acknowledge that the primary goal of triage is to determine which patients require urgent access to care rather than to predict in-hospital mortality. Nonetheless, using in-hospital mortality as a surrogate marker enables researchers to evaluate the possibility of undertriage. Third, aside from mobility status, we did not consider other determinants of frailty. Moreover, frailty was not examined by form or severity (e.g., chronic or acute); thus, bias may have been introduced. However, because triage in the ED aims to prioritise incoming patients and assign them a triage acuity level through a brief, focused assessment, a thorough frailty screening may not be ideal in such as context. Fourth, some data were missing; the available case analysis method was employed to handle the data not missing at random. Finally, the significant statistical differences across groups could be derived from the large sample size of the study; their clinical significance may be debatable.

## Conclusions

The ability of the TTAS to predict in-hospital mortality decreases with patient age. Although frailty as mobility status was associated with in-hospital mortality, its addition to the TTAS only slightly improved the accuracy with which in-hospital mortality in older adult patients presenting to EDs was predicted.

## Supplementary Information

**Additional file 1.**

**Additional file 2.**

## Data Availability

All data relevant to the study are included in the article or uploaded as supplementary information.

## Abbreviations

ED: Emergency department
TTAS: Taiwan Triage and Acuity Scale
ICU: Intensive care unit
AUROC: Area under the receiver operating characteristics curve
CTAS: Canadian Triage and Acuity Scale
SD: Standard deviation
NHI: National Health Insurance
