# Supplementary material for: Using mobility status as a frailty indicator to improve the accuracy of a computerised five-level triage system among older patients in the emergency department
Source: BMC Emerg Med. 2022 May 19;22:86. doi: 10.1186/s12873-022-00646-0 (PMC9118587; doi:10.1186/s12873-022-00646-0)
Supplement: Supplementary file 1 — Additional file 1. [file 12873_2022_646_MOESM1_ESM.pdf]

Risk of adult falling form (translated from Chinese)<br>Total point of 5 indicating high risk of falling.		
Category	Point	Status
Activity and mobility	0<br>1	☐ Normal<br>☐ Difficulty balancing ☐ Unsteady gait ☐ Use of mobility aid<br>☐ Poor muscle in the lower limbs
Use of medications that increase risk of falling	0<br>1	☐ No<br>☐ Yes
History of fall	0<br>2	☐ No<br>☐ Experienced a fall within one year
Dizziness or weakness condition	0<br>2	☐ No<br>☐ Feeling vertigo, dizzy, or weak within one week
Use of tube or monitoring system	0<br>1	☐ No<br>☐ IV ☐ CVP ☐ Chest tube ☐ Foley ☐ Oxygen<br>☐ Physiologic monitor ☐ Infusion pump ☐ EKG ☐ Others
Frequently getting out of bed to go to the toilet or need assistance to go to the toilet	0<br>2	☐ No<br>☐ ≥10 times per day (including pee or poo)<br>☐ Diarrhea (≥3 passages per day)<br>☐ Nocturia ≥2 times per day<br>☐ Feel like going to the toilet more frequently than before hospitalization<br>☐ Need assistance to get out of bed to go to the toilet
