# Supplementary material for: Using mobility status as a frailty indicator to improve the accuracy of a computerised five-level triage system among older patients in the emergency department
Source: BMC Emerg Med. 2022 May 19;22:86. doi: 10.1186/s12873-022-00646-0 (PMC9118587; doi:10.1186/s12873-022-00646-0)
Supplement: Supplementary file 2 — Additional file 2. [file 12873_2022_646_MOESM2_ESM.pdf]

Supplemental Table 1. Most common chief complaints by age group

	Total (N = 265,219)	Age group			
		18–39 (N = 79,860)	40–64 (N = 105,246)	65–84 (N = 64,104)	≥85 (N = 16,009)
1	Abdominal pain (11.3%)	Abdominal pain (12.8%)	Abdominal pain (12.2%)	Abdominal pain (8.8%)	Shortness of breath (13.4%)
2	Fever/Chills (6.0%)	Fever/Chills (6.4%)	Chest pain/Chest tightness (6.0%)	Shortness of breath (8.4%)	Abdominal pain (7.8%)
3	Chest pain/Chest tightness (5.2%)	Pregnancy problem (5.1%)	Fever/Chills (5.2%)	Fever/Chills (6.4%)	Fever/Chills (7.6%)
4	Shortness of breath (4.7%)	Nausea/Vomiting (3.7%)	Vertigo/Dizziness (4.6%)	Chest pain/Chest tightness (6.3%)	Chest pain/Chest tightness (5.8%)
5	Vertigo/Dizziness (4.3%)	Blunt lower limb trauma (3. 4%)	Shortness of breath (3.8%)	Vertigo/Dizziness (6.1%)	Vertigo/Dizziness (4.3%)
6	Local redness and swelling (3.2%)	Laceration and abrasion of upper limbs (3.3%)	Local redness and swelling (3.7%)	Local redness and swelling (3.2%)	Weakness generalized (3.8%)
7	Nausea/Vomiting (2.7%)	Chest pain/Chest tightness (3.1%)	Lumbago (3.1%)	Weakness generalized (3.1%)	Device issue (3.3%)
8	Blunt lower limb trauma (2.4%)	Contusion of upper limbs (2.8%)	Nausea/Vomiting (2.2%)	Device issue (2.9%)	Stool bloody/Stool black (3.3%)
9	Lumbago (2.2%)	Local redness and swelling (2.7%)	Headache (2.2%)	Stool bloody/Stool black (2.5%)	Altered state of consciousness (3.1%)
10	Headache (2.0%)	Vertigo/Dizziness (2.6%)	Blunt lower limb trauma (2.0%)	Nausea/Vomiting (2.3%)	Local redness and swelling (3.0%)
